# Effect of Ice Bag Application to Femoral Region on Pain in Patients Undergoing Percutaneous Coronary Intervention

**DOI:** 10.1155/2017/6594782

**Published:** 2017-05-28

**Authors:** Sevda Korkut Bayındır, Gülsüm Nihal Çürük, Abdurrahman Oguzhan

**Affiliations:** ^1^Department of Nursing, Faculty of Health Sciences, Erciyes University, Kayseri, Turkey; ^2^Department of Nursing, Faculty of Health Sciences, İzmir University of Economics, İzmir, Turkey; ^3^Department of Cardiology, Faculty of Medicine, Erciyes University, Kayseri, Turkey

## Abstract

**Aims:**

The aim of this study is to determine the pain reduction effectiveness of ice bag applications to the femoral region in patients undergoing percutaneous coronary intervention.

**Material and Methods:**

A randomized controlled trial with repeated measures and two-group design. The study was completed with a total of 104 patients who met the inclusion criteria: 52 each in the experimental group and the control group. The pain experienced by the patients was evaluated before and during removal and again while the nurse applied pressure on the catheter site after removal. The NRS scores were identified as NRS1, NRS2, and NRS3 for the three assessment, respectively.

**Results:**

The NRS1 score was similar between the two groups. It increased at the 2nd measurement, and a statistically significant difference was determined between the two groups (4.0 (3.0-4.0) in the experimental group and 6.0 (4.0-7.0) in the control group) (*p* < 0.001).

**Conclusions:**

The results of the study revealed that ice bag application to femoral region was effective in reducing pain induced by femoral catheter removal in patients undergoing percutaneous coronary intervention. Local ice bag application may therefore be recommended as a nursing intervention for pain control in such cases.

## 1. Introduction

Although several arterial access routes may be employed during percutaneous coronary intervention (PCI), the femoral arterial site has been the most commonly used [1]. However, during femoral artery interventions, many patients experience pain and discomfort during the removal of catheters previously inserted into the femoral region [2, 3]. Previous literature also provides evidence that both vasovagal responses and local vascular complications may develop when the pain induced by catheter removal is not effectively controlled [3–5]; therefore, it is important to reduce the pain experienced by patients undergoing this procedure [6].

Pain caused by the removal of a femoral catheter may be controlled by using pharmacological methods such as treatment with morphine sulfate or lidocaine infiltration [2, 3]. However, it has also been observed that such approaches may cause complications such as increased bleeding, laceration of catheters, infection, and temporary nerve injury [7–9]. Pain may also be controlled using nonpharmacological methods, which are patient-specific and aim to establish empathic communication with healthcare staff [10–13]. Nonpharmacological methods used for pain control provided positive effects such as reduced anxiety and psychological support; notably, patients have expressed satisfaction with such nonpharmacological methods [14].

Cold application is a nonpharmacological method of pain control [6]. Being one of the oldest and easiest forms of treatment, cold application increases the threshold of pain and reduces the conduction velocity of nerve fibres transmitting pain stimuli from the peripheral to the central nervous system [15]. Demir and Khorshid [10] investigated the effect of cold application upon pain caused by the removal of a chest tube and revealed that cold application reduced pain intensity and delayed patient requests for a second analgesic. In a further study by Ertuğ and Ülker [11], cold application was highly effective in reducing the pain caused by chest tube removal. Cold application also confers other advantages, such as ease of application, the lack of serious side effects, and low cost [16].

## 2. Aim and Hypothesis of the Study

This study aimed to determine the effect of cold application to the femoral region upon pain levels of patients receiving PCI. Our hypothesis was that cold application would reduce (1) the pain score associated with removal of a femoral catheter after PCI, (2) the behavioural responses of discomfort associated with removal of a femoral catheter after PCI, and (3) the incidence of adverse reactions associated with removal of a femoral catheter after PCI.

## 3. Methods

The study was conducted as a randomized interventional trial between September 2013 and May 2014 at the Yılmaz-Mehmet Öztaşkın Heart and Vascular Hospital located in Kayseri, Turkey. Patients were divided into control and experimental groups by computer randomization. Eligible patients were informed both verbally and in writing, and their written informed consent was obtained. To conduct the study, the necessary written permissions were obtained from both the Erciyes University Clinical Trials Ethics Committee and Erciyes University Hospital (clinical registration number: NCT03131271). The study was approved by the Erciyes University Ethics Committee (2013/513).

### 3.1. Sample

The study was conducted on patients with their consent. Inclusion criteria were as follows: able to speak and understand Turkish, over 18 years of age, about to undergo a femoral intervention to insert a single catheter in their femoral region, unimpaired time and place orientation with no psychiatric disorders or no visual or hearing problem, no cold allergy, normal vital signs, and avoidance of analgesic treatment prior to catheter removal.

### 3.2. Calculation of Sample Size

The sample size was designed using NRS (Numerical Rating Scale) scores ranging from 0 to 10 with a minimum difference of two points between the control and experimental groups at a confidence level of *α* = 0.95 with a power of 0.9. As a consequence, the sample size was determined as 52 patients for each group.

**Figure 1:**
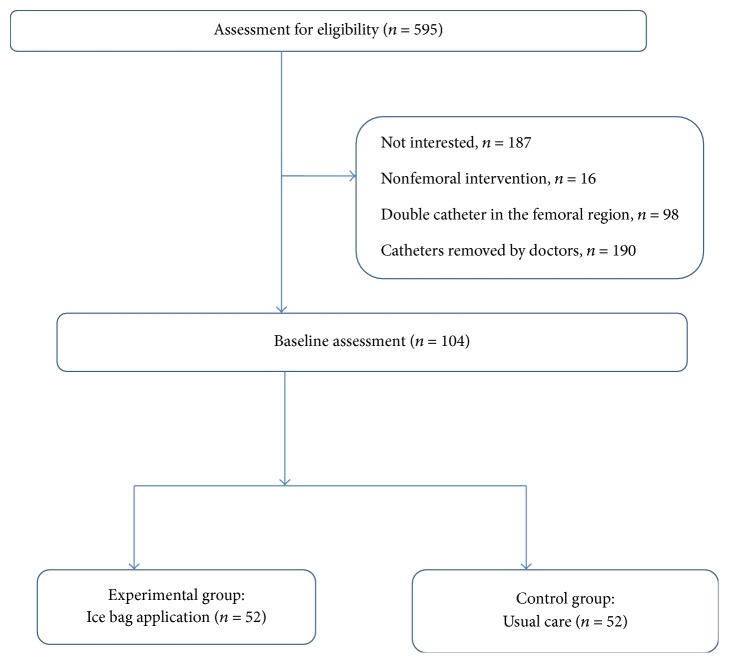
Flowchart of the randomized controlled trial.

The study was completed with 104 patients, who met the inclusion criteria between September 2013 and May 2014.  Figure 1 shows a sample diagram of the study.

### 3.3. Experimental Group

In the experimental group, the researcher provided a cold application for 20 minutes by placing an ice bag to the site of the femoral catheter. Immediately after its removal, the responsible nurse removed the catheter. A neutral instruction set was used on each patient prior to application of the ice pack. Patients in the experimental group were told that they may or may not experience pain during the catheter removal. The patients were also told that the aim of the study was to measure the effect of ice bag application upon pain during catheter removal and that ice pack application may or may not be effective in terms of their own pain.

### 3.4. Control Group

 The control group received the standard clinic procedure. Each control patient was informed that some patients may experience pain during catheter removal and that they may or may not experience pain. Patients were also told that their pain levels would be measured during catheter removal.

In all patients (control and experimental groups), the femoral catheter was removed four hours after PCI by a male nurse responsible for catheter removal in the clinic. As general practice, the clinic did not employ pharmacological (analgesics such as acetaminophen, opioid, or systemic analgesic) or nonpharmacological methods to prevent pain during catheter removal.

### 3.5. Measures

Data were collected by only one researcher using a Patient Identification Form and a Numerical Rating Scale.

### 3.6. Patient Identification Form

After reviewing the related literature, the researcher prepared a 15-question Patient Identification Form, which included sociodemographic characteristics (such as age, education, and gender) and PCI-related characteristics (such as previous percutaneous coronary intervention, catheter size, time to hemostasis, and complications) [10, 11, 17]. The researcher completed the Patient Identification Form by conducting face-to-face interviews with patients following PCI and also by reviewing patient files. Patients behavioural responses (e.g., grimace, eyes closed) during catheter removal were observed and recorded on a preprepared checklist by the researcher. In addition, local vascular complications (such as bleeding and hematoma) were recorded as “yes” or “no.” The researcher also measured the vital signs of each patient immediately before and after catheter removal, examined each patient's catheter zone for any signs of bleeding, hematoma, ecchymosis, and other complications, and recorded these on a checklist.

### 3.7. Numerical Rating Scale

The NRS is a 10 cm tabulated ruler and subjects were asked to indicate their pain on a scale of 0 (no pain) to 10 (the worst pain the patient had ever experienced). The validity of the NRS was examined by Ferreria-Valente et al. [18]. Patients in both experimental and control groups were informed that they might feel pain during catheter removal, and it was explained that the aim of the study was to determine the level of the pain the patients experienced. Both patient groups were informed that the NRS would be used in order to evaluate their pain level before, during, and after catheter removal. They were instructed on how to use the NRS, which involved indicating their level of pain on a 10 cm ruler, ranging from 0 (no pain) to 10 (worst pain). They were also asked to mark the score indicating the level of pain they were currently experiencing.

Pain was evaluated three times: immediately prior to catheter removal, during catheter removal, and while the nurse applied pressure on catheter site within the first minute after removal. NRS scores were identified as NRS1, NRS2, and NRS3, for each evaluation, respectively.  Table 1 shows the application of this system to the patients in the experimental and control groups.

## 4. Statistical Analyses

The data were analysed with the SPSS 15.0 statistical software package. The Shapiro-Wilks test was employed to determine whether data were normally distributed. As the distribution was not normal, nonparametric tests were used. Categorical variables were compared with the Chi-square exact test and intergroup comparisons of numeric variables were performed by the Mann-Whitney* U* test. Comparisons of repeated measurements were performed using the Wilcoxon test and Friedman analysis for two and three measurements, respectively. Post hoc analysis was conducted on NRS scores between groups and between each of the three time points. Descriptive statistics are shown as means, standard deviations, medians (25%–75%), and percentage values.

## 5. Results

This study was completed with a total of 104 patients (52 patients in each of the control and experimental groups). The majority of the patients were males, aged between 51 and 70 years, and diagnosed with coronary artery disease. More than half had previously undergone PCI (experimental group: 55.8%; control group: 63.5%) and almost all (experimental group: 93.1%; control group: 93.9%) reported experiencing pain during catheter removal. The groups were similar in terms of their demographic characteristics (*p* > 0.05; Table 2). In the majority of cases, in both the control and experimental groups, 7 French (Fr) catheters were used and remained in situ for approximately four hours. Following catheter removal, the median values for application of pressure on the insertion site were 6.1 minutes (range: 4.6–7.4) and 9.1 minutes (range: 7.2–11.3) minutes for the experimental and control groups, respectively (*p* < 0.001) (Table 3). No cardiac complications (arrhythmia, ischaemia, and vasovagal reaction) emerged during this study.

No complications occurred in the experimental group following catheter removal, whereas bleeding complications developed in 9.6% of the control group (*p* = 0.05) (Table 3). During catheter removal, it was observed that the majority of patients in the control group showed some reactions, such as wincing, fist clenching, intervening with hands, biting fingers or lips, grinding teeth, and crying or moaning, and the difference between the control and experimental groups in terms of these pain reactions was statistically significant (*p* < 0.001; Table 3). Furthermore, a statistically significant difference (*p* < 0.001; Table 4) was observed between the median values of NRS scores in terms of the observational periods. In our advanced analysis, it was determined that the median values of the NRS scores increased in both the control and experimental groups from NRS1 to NRS2, which was more prominent in the control group (*p* < 0.001). This difference remained at the 3rd observation (Table 4).

## 6. Discussion

In their study, Puntillo and Ley, [19] examined pain responses during six common medical procedures and reported that uncomfortable pain was experienced during catheter removal. Numerous studies can be found in the literature relating to the pain experienced by PCI patients following catheter removal [2, 20, 21]. In the present study, it was observed that all of our patients experienced pain, with an average intensity of 4 (range: 3.0–6.0) (NRS2 and NRS3), which is consistent with previous literature [2].

Pain adversely affects the recovery process in patients, by causing anxiety and fatigue [15]. Pain causes the release of catecholamines and thereby increases both cardiac workload and oxygen consumption, which may lead to the development of arrhythmias, ischaemia, acute cardiac failure, and acute myocardial infarction in patients with coronary artery disease [22]. Therefore, it is considered essential to take into account the pain experienced by patients during catheter removal following PCI, in order to examine their feelings of pain, determine their pain level with appropriate scales, and administer relevant nursing interventions for relieving their pain.

Pain causes analgesic consumption and increases the additional analgesic needs of patients [22]. Previous studies also reported that analgesic therapy is applied or additional analgesic therapies are required in order to control the pain associated with removal of the femoral arterial catheter following PCI [2, 23]. Because pharmacological methods of pain management are associated with side effects, nonpharmacological therapies should be considered as alternatives. Therefore, it appears to be important to control the pain before it starts during invasive interventions that cause pain experience in patients and to take nonpharmacological methods such as cold application into account in pain control to reduce the patient's analgesic requirements.

Among the nonpharmacological methods of pain, cold application increases analgesic efficacy by enhancing the threshold of pain, reducing the conduction velocity of the unmyelinated small-diameter nerve fibres, which are responsible for transmitting pain stimuli from the peripheral to the central nervous system [15]. In the present study, although both study groups experienced pain, the pain level experienced by the experimental group was lower than that of the control group (NRS2 and NRS3), and this difference was statistically significant (*p* < 0.001) (Table 4). This result confirms our hypothesis that “ice bag application to the femoral region in patients undergoing PCI is effective in reducing pain during catheter removal.” Based on this finding, we can assert that cold application is an important nonpharmacological nursing initiative in pain management.

Insufficient pain control during catheter removal can cause vasovagal reactions and/or vascular complications [24]. There exist cases in the literature that vasovagal reaction develops due to the pain experienced by patients during removal of the femoral arterial catheter following PCI [2, 24]. Vasovagal reactions may cause irreversible shock and even death, if not effectively treated. In addition, it causes anxiety in patients, prolongation of the length of hospital stays, additional work load, and increases in overall costs. In the clinic, in which our study was conducted, no pharmacological or nonpharmacological interventions regarding the pain are performed prior to removal of the femoral catheter and patients' pain experiences are not questioned. Therefore, it can be stated that it is necessary and important to create awareness in nurses that the pain, which might develop due to removal of the femoral catheter following PCI, does trigger the vasovagal reaction.

In our study, it was detected that no bleeding complications developed in our experimental group (*p* = 0.05) in addition to the shorter hemostasis duration (*p* < 0.001). This finding suggests that early hemostasis develops through ice bag application in those patients in the experimental group so that the bleeding can be taken under control. In this context, this finding can also be considered clinically significant. Cold application controls bleeding via the vasoconstriction of arterioles and also increases coagulation of he blood by reducing flow speed and increasing viscosity. Thus, increasing blood coagulation and reducing capillary permeability and metabolic requirements provide easy options with which to control bleeding [25–27].

In the literature, pain severity and complications have been reported to increase with the size of the catheters used for PCI [23, 24]. For example, Zeller et al. [28] reported complication-free discharge among patients, during transfemoral angiography with 4 Fr catheters three hours after removal of the catheter. In the present study, the majority of patients in the experimental and control groups received 7 Fr catheters, which may have been responsible for the relatively high pain mean scores.

Personal reactions to pain are variable [15]. Puntillo and Ley, [19] investigated pain behaviours during six common medical procedures and observed that patients responded in a variety of ways, including grimace (42.8%), closure of eyes (33.7%), rigid reactions (26.8%), and wincing and verbal complaints (23.7%). The observed behavioural responses recorded in the present study were similar to those reported by Puntillo and Ley [19], although our response rates were lower in the group treated with cold application.

## 7. Limitations and Future Research

This study was conducted in a local Heart Hospital located in Kayseri; therefore, its results may not be generalized to other settings. A second limitation is the lack of a sham treatment for the control group or at minimum equal time with the nurse, which the experimental group experienced during the ice bag application. The absence of the nurse during ice pack application might reduce pain expectations and responses of the patients; therefore, it might limit the findings. However, we cannot exclude the possibility of a Hawthorne effect in our subjects, because they were aware of the study design. Given the ethical requirements for informed consent, the Hawthorne effect cannot be tested. Furthermore, the present study did not question whether or not the patients were satisfied with the application. It is recommended to conduct future randomized controlled studies that compare the efficiency of ice pack application in pain, experienced during the catheter removal, by using different nonpharmacological methods.

## 8. Conclusion

This is the first study to investigate the effect of cold application upon the pain caused by removal of a femoral artery catheter following PCI. The results of this study revealed that cold application before catheter removal reduced pain intensity and limited local vascular complications.

## Figures and Tables

**Table 1:** Applications to the patients in the experimental and control groups.

	Experimental group	Control group
Before the catheter removal	(i) The patients were informed both verbally and in writing (ii) Their written informed consent was obtained (iii) The patient identification form was completed (iv) Cold application was performed for 20 minutes by placing an ice bag over the entry site of femoral catheter (v) NRS1 was evaluated (vi) The patient's vital signs were measured and recorded	(i) The patients were informed both verbally and in writing (ii) Their written informed consent was obtained (iii) The patient identification form was completed (iv) NRS1 was evaluated (v) The patient's vital signs were measured and recorded

During the catheter removal	(i) NRS2 was evaluated (ii) Behavioural responses given by the patient during the removal of catheter were observed and recorded	(i) NRS2 was evaluated (ii) Behavioral responses given by the patient during the removal of catheter were observed and recorded

After the catheter removal	(i) NRS3 was evaluated (ii) The patient's vital signs were measured and recorded (iii) The pressure time was recorded (iv) The patient's catheter zone was examined for complications	(i) NRS3 was evaluated (ii) The patient's vital signs were measured and recorded (iii) The pressure time was recorded (iv) The patient's catheter zone was examined for complications

**Table 2:** Demographic variables of the experimental and control groups.

Variable	Categories	Experimental number (%)	Control number (%)	*p*
Age	(*X* ± SD)	62.1 ± 13.4	61.6 ± 12.7	0.852

Gender	Female Male	13 (25.0) 39 (75.0)	14 (26.9) 38 (73.1)	1.000

Diagnosis^*∗*^	ACS CAD AMI AP Others	15 (28.3) 27 (50.9) 8 (15.1) 2 (3.8) 1 (1.9)	19 (35.9) 22 (41.5) 4 (7.5) 3 (5.7) 5 (9.4)	0.269

Type of chronic diseases	DM HT CAD COPD Others	16 (30.3) 22 (41.5) 7 (13.2) 4 (7.5) 4 (7.5)	18 (28.7) 30 (47.6) 5 (7.9) 5 (7.9) 5 (7.9)	0.902

Previous percutaneous coronary intervention	Yes No	29 (55.8) 23 (44.2)	33 (63.5) 19 (36.5)	0.424

Previous experienced pain	Yes No	27 (93.1) 2 (6.9)	31 (93.9) 2 (6.1)	0.818

^*∗*^Given more than one answer, percentages were shown via *n*; ACS: acute coronary syndrome; CAD: coronary artery disease; AMI: acute myocardial infarction; AP: angina pectoris; DM: diabetes mellitus; HT: hypertension; COPD: chronic obstructive pulmonary disease.

**Table 3:** Clinical characteristics of the experimental and control groups.

Variable	Categories	Experimental number (%)	Control number (%)	*p*
Mean catheter size (French)	6 Fr.	3 (5.8)	6 (11.5)	0.295
	7 Fr.	48 (92.3)	43 (82.7)	
	8 Fr.	1 (1.9)	3 (5.8)	

Time to hemostasis (minutes) median (25%–75%)		6.1 (4.6–7.4)	9.1 (7.2–11.3)	<0.001

Complication	Yes^*∗*^	0 (0.00)	5 (9.6)	0.057
	No	52 (100.0)	47 (90.4)	

Response to catheter removal^*∗∗*^	Grimace	41 (78.8)	42 (80.7)	<0.001
	Eyes closed	31 (59.6)	37 (71.1)	
	Verbal complaint	9 (17.3)	15 (28.8)	
	Unable to assess	9 (17.3)	9 (17.3)	
	Others^*∗∗∗*^	4 (7.6)	50 (96.1)	

^*∗*^Only bleeding developed as a complication. ^*∗∗*^Given more than one answer, percentages were shown via *n*. ^*∗∗∗*^Rigid, wincing, hesitation, clenching the fists, intervention with the hand, biting a finger, biting the lips, crying, and moaning.

**Table 4:** Comparison of NRS scores of the patients in the experimental and control groups.

	Groups	Experimental	Control	*p*
Median (25%–75%)	NRS1	0.0 (0.0-0.0)^a^	0.0 (0.0-0.0)^a^	1.00
	NRS2	4.0 (3.0–4.0)^b^	6.0 (4.0–7.0)^b^	<0.001
	NRS3	4.0 (3.0–4.7)^b^	6.0 (4.0–7.0)^b^	<0.001
	*p*	<0.001	<0.001	

Mean ± SD	NRS1	0.1 ± 0.4^a^	0.1 ± 0.4^a^	1.00
	NRS2	3.6 ± 1.4^b^	5.6 ± 2.3^b^	<0.001
	NRS3	3.8 ± 1.4^b^	5.5 ± 2.1^b^	<0.001
	*p*	<0.001	<0.001	

^*∗*^The same letters signified no difference and different letters signified the presence of both between-groups and within-group differences.
